# Perceptions on the sexual harassment of female nurses in a state hospital in Sri Lanka: a qualitative study

**DOI:** 10.1080/16549716.2018.1560587

**Published:** 2019-02-26

**Authors:** Emma A. Adams, Elisabeth Darj, Kumudu Wijewardene, Jennifer J. Infanti

**Affiliations:** a Department of Public Health and Nursing, NTNU, Norwegian University of Science and Technology, Trondheim, Norway; b Strategic Initiatives, Ontario Shores Centre for Mental Health Sciences, Whitby, Ontario, Canada; c Department of Obstetrics and Gynecology, St Olavs University Hospital, Trondheim, Norway; d Department of Women’s and Children’s Health, Uppsala University, Uppsala, Sweden; e Department of Community Medicine Health, Faculty of Medical Sciences, University of Sri Jayewardenepura, Nugegoda, Sri Lanka

**Keywords:** Sexual harassment, occupational health, nurses, perception, qualitative research, attitude of health personnel

## Abstract

**Background**: Sexual harassment occurs within the nursing profession globally, challenging the health and safety of nurses and the quality and efficiency of health systems. In Sri Lanka, no studies have explored this issue in the health sector; however, female employees face sexual harassment in other workplace settings.

**Objective**: To explore female nurses’ perceptions of workplace sexual harassment in a large state hospital in Sri Lanka.

**Methods**: This is a qualitative study conducted in an urban, mainly Buddhist and Singhalese context. We invited all female senior and ward nurses working in the hospital to participate in the study. We conducted individual in-depth interviews with four senior nurses and focus group discussions with 29 nurses in three groups.

**Results**: The nurses described a variety of perceived forms of sexual harassment in the hospital. They discussed patient-perpetrated incidents as the most threatening and the clearest to identify compared with incidents involving doctors and other co-workers. There was significant ambiguity regarding sexual consent and coercion in relationships between female nurses and male doctors, which were described as holding potential for exploitation or harassment. The nurses reported that typical reactions to sexual harassment were passive. Alternatively, they described encountering inaction or victim blaming when they attempted to formally report incidents. They perceived that workplace sexual harassment has contributed to negative societal attitudes about the nursing profession and discussed various informal strategies, such as working in teams, to protect themselves from sexual harassment in the hospital.

**Conclusions**: Sexual harassment was a perceived workplace concern for nurses in this hospital. To develop effective local prevention and intervention responses, further research is required to determine the magnitude of the problem and explore differences in responses to and consequences of sexual harassment based on perpetrator type and intent, and personal vulnerabilities of the victims, among other factors.

## Background

Workplace sexual harassment has been referred to as any unwanted sex-related behaviour at work appraised by the recipient as offensive, exceeding her (or his) resources, or threatening her well-being [1]. It is becoming increasingly clear that sexual harassment is a global workplace hazard linked to a range of short- and long-term physical and psychological health consequences [2,3]. Workplace sexual harassment also creates unacceptable working conditions that undermine the recruitment and productivity of employees.

In the nursing profession, sexual harassment occurs across the globe. Nurses are among the occupation groups most likely to experience offensive sexual behaviours at work [4–9], although prevalence rates differ considerably by country, workplace setting, and research methodology. Nurses face harassment from both patients and colleagues. The type of perpetrator, intentionality of the perpetrator, and relationship between the nurse and perpetrator are associated with differing adverse mental health outcomes and other aspects of well-being [10]. In addition to threatening the health of individual nurses [5,11–13], workplace sexual harassment can affect the quality of patient care and reduce the efficiency and quality of the entire health system.

Given the serious nature of the concerns about sexual harassment in nursing, the phenomenon has been well studied in high-income contexts [6–8]. Corresponding prevention efforts and policy guidance to reduce its occurrence are also well established in the healthcare settings in many of these countries [14]. It is challenging to translate successful models for prevention and intervention from one health setting to another as each organization is unique and sexual harassment is not necessarily a predictable workplace health problem. Therefore, it is important to explore local nuances of the problem.

In Sri Lanka, working women are subjected to different forms of gender-based violence regardless of their socioeconomic status [15]. Sexual harassment is a criminal offence under the Penal Code in Sri Lanka. The perpetrator of sexual harassment may be punished with imprisonment up to a term of five years, a fine, or both [16]. Despite this strict policy, sexual harassment is rarely reported [12]. Studies on the prevalence of sexual harassment within Sri Lanka are limited, but 90% of Sri Lankan women have reported incidents on buses and trams at some point in their lives, and half of these women (50%) were on their way to work when the incident occurred [17]. No prior studies exist on the sexual harassment of healthcare providers.

Perceptions and experiences of the phenomenon of sexual harassment, as well as its influencing factors or determinants, vary across and within countries [3,18–20]. A better understanding of the problem in specific South Asian countries and workplace settings is required to ensure effective prevention efforts and interventions, and an effective workforce. The current gaps in research on this topic are an impediment to improving the response of local health sectors to this serious health concern. This study fills some of these knowledge gaps, by exploring female nurses’ perceptions of sexual harassment in a large state hospital in Sri Lanka.

## Methods

### Study design

A qualitative design was chosen as an appropriate and useful way to explore an issue as complex, multifaceted, and understudied as nurses’ perceptions of sexual harassment in Sri Lanka.

### Study setting

Sri Lanka’s health system provides free and accessible services for all citizens from primary to tertiary levels [21,22]. The study hospital is integrated in the system, and services a primarily urban-dwelling and Buddhist population of Singhalese ethnicity and language. The Singhalese population represents approximately 80% of Sri Lanka’s total population.

### Data collection

In 2016, the research team sought permission from the hospital director and head matron to conduct the study among female nurses. Following this, the head matron provided the research team with a list of all senior nurses working in the hospital (n = 8) and assisted in making initial contact with them. We invited senior nurses by telephone to participate in in-depth interviews (IDIs), and four IDIs were conducted, lasting one hour each. The senior nurses were asked to allow the nurses under their supervision to participate in focus group discussions (FGDs). Thereafter, three FGDs were held in a secure on-site location with a total of 29 nurses representing all wards in the hospital. The FGDs lasted between one and two hours each. The principles of voluntary and informed consent were explained at the start, and information about anonymization of the data was provided.

The first and third authors conducted the IDIs and FGDs in Sinhala, English, or both languages. An independent person assisted with translations of the audio-recordings to English where necessary, and English transcripts were also created, incorporating observation notes taken during the IDIs and FGDs.

### Study participants

Two of the eight senior nurses in the hospital did not participate in the study because of prior obligations during the data collection period, and two chose not to participate. Four senior nurses participated in the IDIs and they were asked to invite between five and eight nurses each from their wards to participate in the FGDs. We requested that the ward nurses be selected purposefully to represent different divisions in the hospital, experience, role, age, and seniority. The study was limited to female nurses as they represent the vast majority of the total nursing workforce in Sri Lanka. Nurses had to currently work at the hospital to enrol in the study.

### Interview guide

The terminology used and categorizations of sexual harassment are often contested, creating a large degree of ambiguity and self-determination regarding perceived sexual harassment in the literature on the topic [3]. This study took guidance from the International Labour Organization’s (ILO’s) recommendations for studying sexual harassment in the workplace [2]. Nurses were prompted in the interviews to consider four types of sexual harassment: verbal, gestural, physical, and visual. Examples were given to clarify the typology as needed.

We used a similar interview guide for both the IDIs and FGDs. Over the course of data collection, minor adjustments were made to the guides. The only notable variation between the initial and final guides reflected the tendency for nurses to discuss incidents based on the perpetrator, rather than the subtype of sexual harassment. Incidents of sexual harassment could not always be distinctly categorized into one subtype, and thus the guides were changed to capture the perspectives of participants.

### Data analysis

The first author reviewed each transcript and produced a detailed written summary, which also incorporated written notes from IDI and FGD observations. The summaries were reviewed by the entire research team. Once the team felt that thematic saturation had been reached, they stopped collecting data. The authorship team then engaged in content and thematic analysis of the data, influenced by the four stages of theme development suggested by Vaismoradi et al. [23]. Simultaneously, the first author coded the complete set of transcripts, using NVivo 11 software to organize the data. After the first IDI and FGD were each coded, the last author reviewed the initial code list and added new codes. The first author then returned to the complete set of transcripts to review and recode them. Comparisons between the codes and feedback from all authors on the themes in the data as a whole led to subsequent revisions of the codebook. This iterative and reflective process led to the generation of secondary and final themes.

### Research reflexivity

All researchers were female. Two were medical doctors and two were non-clinical researchers, and each had previous experience conducting qualitative research. One medical doctor and one non-clinical researcher conducted the interviews. Before commencing the study, the researchers discussed their preconceptions on the topic and concluded that they expected sexual harassment would exist within the workplace and would be underreported. However, no prior expectations were mentioned involving likely types, perpetrators, or characteristics of sexual harassment.

### Ethics

The Sri Lankan Ethics Review Committee formally approved a questionnaire-based study which preceded this research in 2015, and the addition of this qualitative component in 2016 (application number 17/15). An application was also submitted to the Regional Committee for Medical and Health Research Ethics in Norway (reference 2016/2157/REK midt). As no patients were interviewed, the Norwegian board determined the study to fall outside their mandate, and suitable to be implemented and published without the committee’s formal approval. The Norwegian Center for Research Data was consulted and concluded that the study did not require their ethical approval.

A number of ethical considerations guided the collection of data. The topic of sexual harassment, like all discussions related to sexuality, is considered sensitive and taboo in Sri Lanka. Therefore, the topic guides for the IDIs and FGDs focused on the general existence of sexual harassment, rather than personal experiences. The topic was introduced using more culturally acceptable words and concepts like ‘bad behaviours’ rather than ‘sexual behaviours’. No individual characteristics of the nurses are presented in this paper and the hospital name and location are also not disclosed to ensure anonymity. To mitigate potential power imbalances linked to workplace roles and hierarchies, the head nurses were interviewed individually and independently from the nursing staff under their supervision.

## Results

We divided the study findings into three main themes: incidents of sexual harassment; consensual relationships; and reactions, responses, and consequences to sexual harassment.

### Incidents of sexual harassment

A variety of incidents were perceived as the occurrence of sexual harassment in the hospital. The nurses did not classify them into discrete categories or types of harassment, but rather spoke of a combination of types, for example, physical harassment alongside verbal or non-verbal harassment. The nurses differentiated between incidents perpetrated by male patients and male co-workers.

#### Patients

Sexual harassment perpetrated by patients was described as common in male wards, relatively clear for nurses to identify, and burdensome for nurses, especially during night shifts. Various incidents of flashing were described, wherein patients exposed their private parts inappropriately to nurses who were trying to perform their care-giving responsibilities in the night. The nurses shared some of their experiences of harassment perpetrated by male patients:

Some of the patients remove their clothes … just to harass the nurses at night because there are only a few nurses at the station in the night.

At night, we had to give the night dose of medicine, sometimes through the existing IV in the hand. When we went to do that, the patient would stroke you and get vulgar. They would touch you and try to get physical and they put their phone numbers into the books that we carry. In orthopaedics you get patients who are there for a long time, and they say to you, “I haven’t had a body wash in a real long time”. They come onto you really strong and touch you inappropriately. They make dirty, vulgar, comments. They say “I was waiting for you to do this and that for me”. Make disturbing comments like that.

The nurses also described scenarios in which patients and their visitors harassed them on their wards:

When patients are very sick, they are allowed to have someone stay with them on the ward. Patients are kept together, so two or three of these bystanders can gang up and inform the nurse on duty that one of the patients needs something. Then when she goes there, they try to harass her … That is one of the hazards of the nursing career.

Some patients take photographs or make videos of the nurses with their mobile phones and then use them to make pornography [later].

The nurses also discussed how patients harassed them outside their work environments:

They pester the other nurses to get the phone numbers of the nurses they admire and think are pretty.

They stalk us, and give us nuisance calls, and turn up at the hospital when they know we are on night shift.

One nurse described an incident where a patient came to her boarding house and tried to make sexual advances there, explaining:

[The patients] get to know us personally when they stay long periods for their illness and they try to get involved with us then, take advantage of the familiar-feeling relationship.

The nurses also speculated on the roots of patient harassment of female nurses. They perceived the primary reason to be a lack of self-control and sanctions on men’s sexual behaviour:

According to our culture, sexuality is very much controlled and restricted. Many people are unable to control their feelings.

The nurses also discussed other contributing factors including alcohol abuse, a negative upbringing or family background, a sense of power gained through abuse, emulating media depictions, and the availability of technology as a mode of harassment.

#### Doctors

Sexual harassment by male doctors was described as a regular and expected part of work life for female nurses. It was described by the nurses mainly in terms of teasing or joking rather than as physically hazardous or acts with the intent to harm:

It is extremely rare for a doctor to attack a nurse physically … [but] in front of the patients and, in public, they flirt around [with nurses] – cheap flirtations … Some doctors also make vulgar jokes in front of patients and everyone else.

Another nurse explained her perception of a doctor’s intent when harassing her personally at the start of her nursing career:

As soon as I came in – as a new nurse – a doctor called from the general hospital and said, “Oh you have a new voice, what’s your name?” I got afraid and gave my name, and then he started to give me nuisance calls and asking for me by name … That doctor [still] calls us and asks when we are off duty. He uses endearing terms when talking to us – calls us sweetheart. That’s it. He doesn’t pester anybody beyond that.

### Consensual relationships

The nurses raised numerous concerns about consensual relationships between female nurses and male co-workers of various professions in the hospital. Many of the salient words used to describe these relationships had negative connotations, such as ‘affairs’ which left nurses ‘wrecked’, ‘ripped off’, ‘distant’, and ‘ditched’. The nurses were generally uncertain whether these relationships constituted a type of sexual harassment or not, but they spoke about the relative lack of power of nurses and the blurriness of sexual consent in these relationships. Several of their perceived fears and concerns were about the intent of the male parties in entering these relationships, as exemplified by the following anecdotes:

There can be the affairs [by junior doctors] with nurses, but I’m not sure if they are exploitative – we don’t know. We know only they have an affair.

Some doctors just come and say they are interested in us and then ask if we will start an affair with them. They ask for our consent, but if we don’t consent, a lot of the time they use their influence to transfer us out of the ward or hospital.

The nurses suggested that they expected long-term relationships to evolve when they entered into such ‘affairs’ with a doctor. It was their perception, however, that some doctors intended only to participate in what the nurses described as ‘cheap flirtations in the workplace’. The consequences, when a long-term relationship failed to develop, were clearer to explain. The impact was described as long-lasting for the nurses but insignificant for the doctors:

Medical students start affairs with the nurses, then they just dump them and the doctor goes along with his life. There are nurses who have worked a lifetime in nursing and have never got married because a medical student ditched them. So their lives get messed up.

When the intern student completes his training, the nurse gets dumped … Sometimes the doctors hand them over. They go to the next doctor who is coming in and say, “I am finished with her, you can have her”.

### Reactions, consequences, and responses to sexual harassment

The majority of anecdotes shared in the FGDs highlighted that nurses tended to respond passively to incidents of sexual harassment, both as they happened and in the longer term.

#### Immediate reactions

The immediate reactions of individual nurses to incidents of sexual harassment were described as ‘staying quiet’ and ‘doing nothing to draw attention to the situation’. The nurses explained:

People just don’t report it. They are too embarrassed.

Some people don’t report it because they think there will be problems for them as other nurses will think they are of bad character, or people in the family will find out … There is some shyness and they think that it might bring hurt to their homes, and that will bring [other] difficulties.

The nurses discussed other factors and attitudes that contributed to ignoring or otherwise reacting passively to incidents of sexual harassment such as cultural sanctions related to exposure to sexuality, fear of creating further problems at work, and not feeling ‘good enough’ for doctors or other male co-workers in the case of consensual relationships.

Regarding nurses whom did seek formal help or report sexual harassment in the hospital, the available options for assistance were described as filing a formal complaint to the head ward nurse, asking for a change to their place of work or shift times, or speaking to a psychiatric consultant.

A senior nurse shared her efforts to assist junior nurses when they are harassed by patients or doctors:

If problems with patients occur, if there are male nurses or male attendants … I will tell the female nursing officers not to go there [to the patient] too much … [And] if there is any problem with the nursing officer and harassment by a doctor, we … change the nursing officer if possible with her consent to another ward, or change their shifts to reduce the likelihood of meeting the doctor who is harassing her.

Despite the efforts of some of the head nurses, the discussions highlighted that nurses also experienced inaction or blaming when they asked for help or redress following sexual harassment.

#### Degrading the profession

The nurses discussed their perception that their profession has become associated with promiscuity. They expressed that nursing was no longer the ‘noble’ profession it was once considered and was now often described in society as ‘distasteful’. One nurse shared her opinion that doctors contributed to the societal devaluation of nursing when they,

[Flirted with nurses] openly, in front of everyone, [causing] medicine to get a bad reputation. Medicine is not a cheap and immoral thing. It has to have dignity and class.

Another nurse continued:

Society looks at nursing as an immoral career and nurses [themselves] as immoral. A female doing night duty is [now considered] an immoral practice in the Sri Lankan mentality. We have no opportunity or way to turn that view around – even the doctors look at nurses from that point of view.

Another nurse, in the same discussion group, added:

The nursing career is looked down on because the whole of the Sri Lankan community thinks that nurses just give themselves to doctors exactly how the doctors want it. Some in society think that night duty is primarily for sleeping with the doctors. That stigma is there.

Many nurses in the study expressed that these attitudes and perceptions contributed to difficulties in obtaining marriage proposals using the traditional marriage system in Sri Lanka. Suitors assumed the women were exposed to premarital sexuality due to their jobs, and parents of suitors questioned the virginity of nurses. Consequently, nurses explained that their marriages were ‘for love’ because the traditional marriage system was not appropriate. They felt this directly related to sexual harassment experienced in the workplace. They perceived that the degradation of the profession made patients and other males on the hospital wards feel entitled to harass them sexually.

#### Nurses’ informal strategies and suggestions

Nurses discussed informal sexual harassment prevention strategies. They mentioned strategies such as using humour to bring attention to uncomfortable situations when others are nearby, asking for help from their head nurses, and moving around the wards or visiting ‘troublesome’ patients in groups. They discussed how senior nursing colleagues warned younger nurses about the doctors or other co-workers who were known to be ‘bad’. One of the themes that emerged in the discussions was of the senior nurse’s role as protector. Senior nurses not only warned other nurses about doctors, but made it their responsibility to teach junior nurses strategies to handle ‘difficult’ patients, and to say no or ask for help:

I do not think it is part of any [formal] lecture but, in nursing training on the job, the senior tutors and other senior members normally talk with the students about how it [harassment] might happen and how we have to be aware.

The nurses in the study felt that there were no clear hospital-wide guidelines for handling incidents of sexual harassment. They suggested a judgement-free counselling service be made available in hospitals. One of the senior nurses expressed the need for female counselling officers. Other participants felt that counsellors should be available outside the hospital. Overall, the nurses felt that improvements needed to occur in the handling of incidents of sexual harassment within the workplace.

## Discussion

Gender-based sexual harassment of females by male perpetrators in the nursing profession is common. Studies dating back to the 1980s demonstrate the frequency and extent of sexual harassment in nursing, and more recent studies continue to confirm the existence of the phenomenon. Quantitative studies show prevalence rates of sexual harassment within the nursing profession ranging from 30% to 97%, depending on the context [4–9]. The nurses we consulted in Sri Lanka described numerous incidents of sexual harassment that plagued their work. Male co-workers were identified as perpetrators, but the incidents that were described as most threatening and easily identifiable were perpetrated by male patients. The literature on this topic also recognizes a variety of perpetrators of sexual harassment in hospital settings, with patients being the most common [4,5,7,8,10,24–26].

Several studies suggest that nurses’ responses to sexual harassment are generally passive [5,24,25,27]. This is in line with our findings, where the nurses attributed the reasons for their passive reactions to shyness, cultural sanctions about sexual behaviour, fear, and potential victim blaming. Thus our study supports prior research which highlights socialized norms of victim blaming, power dynamics, implications for job security, feelings of shame, and other potential negative repercussions as factors contributing to underreporting [25,27]. Another consideration is that nurses in our context may react passively to sexual harassment owing to a lack of professional vocabulary for and practice in identifying and conceptualizing the phenomenon [28].

### Role-spillover implications

Nurses have occupation and gender roles that may influence the probability of occurrence and experience of workplace sexual harassment. A Japanese study argues that nurses react passively to incidents of sexual harassment because the roles and relationships between nurses and patients are highly regulated [24]. Organizational restrictions, societal norms, regulations, and other sanctions in Sri Lanka may similarly perpetuate passive responses to incidents of sexual harassment.

In Sri Lanka, females have historically been tasked with the role of homemakers. As they have joined the formal employment sector, females have faced the double burden of working while also remaining responsible for all activities in the home. Nursing is often viewed as emotional labour that requires offering some degree of empathy to patients [11]. This empathy and emotional labour can make it easier for patients to blur the lines between work and gender role expectations from nurses [11,28]. This may explain why the nurses in our study described patients as the most frequent perpetrators of sexual harassment, and may also enable male patients to exert their gender power over nurses.

### Intent implications

Sexual harassment is not an accidental workplace problem, but rather involves intentionality. Nurses’ perceptions of the intent behind actions and comments of a sexual nature influence what they consider to be sexual harassment. Our study findings suggest that nurses often dismiss doctors’ sexual comments as jokes intended to lighten the mood of the work environment rather than acts with the intent to harm. Sexual harassment is a subjective experience, and doctors and nurses may have greater rapport and longer working relationships than other co-workers and certainly patients – a factor which was noted in an Australian study as potentially mediating the experience of sexual harassment [29]. In contrast, a quantitative study in Denmark found that patients were more likely to be the perpetrators of sexual harassment, but harassment by colleagues, supervisors, or subordinates was associated with higher mean levels of depressive symptoms for the nurses [10].

We did not explicitly investigate the relative impact of sexual harassment on depressive symptoms or other health outcomes, by perpetrator type or relationship. It may be that the nurses in our study attached particular significance to the alleged ‘consensual affairs’ between female nurses and male doctors in the hospital because ambiguity about sexual consent and coercion in these relationships had a particularly detrimental impact. In future research, it would be prudent to explore nurses’ perceptions of intentional and unintentional sexual harassment by patients and colleagues distinctly.

### Societal implications

Studies have raised the issue of sexual harassment as an occupational hazard for nurses since the mid-1990s [6,9]. Nurses in this study highlighted societal associations of nursing with promiscuity. They felt that nurses are considered overly exposed to sexuality as a consequence of their workplace environment, which has a number of negative implications for the well-being of individual nurses. Nurses were concerned about societal stereotypes of nurses hindering traditional marriage proposals. Marriage in Sri Lanka is still important for the economic and psychological well-being of women [30]. Some families believe that marriage is the primary goal for women’s self-fulfilment. Beyond the impact of societal stigma on individual nurses and their families, negative sociocultural attitudes and views of nursing may impact the likelihood of individuals joining the nursing career. This could be a factor influencing the overall shortage of nurses in Sri Lanka [31].

When trying to address future strategies for reducing sexual harassment in this context, nurses’ perceptions should be considered. The nurses in our study suggest a need for hospital-wide reporting and accountability systems for responding to incidents of sexual harassment. However, they emphasized that formal measures need to be judgement free in order to reduce concerns about potential victim blaming.

### Strengths and limitations

This study begins filling notable knowledge gaps in research evidence on sexual harassment and public health. The inclusion of a diverse research team ensured that the personal biases and motivations of each researcher involved were challenged and considered, and enhanced the overall confirmability of the study. The involvement of multiple researchers increased the availability and accessibility of data, which enriched the interpretation of the findings.

The nurses interviewed were mainly Singhalese Buddhist, which is representative of the geographic setting in which the study was conducted. Similar research should be carried out in other regions of Sri Lanka to explore potential variations in attitudes, points of view, experiences of and reactions to workplace sexual harassment that could be linked to ethnic, cultural, and linguistic background. Similarly, the study should be replicated in other hospitals to obtain broader conclusions about sexual harassment in the nursing profession. Our study explored any experiences of sexual harassment in the workplace. It may prove fruitful for future studies to focus on recent events only and look for age-specific variations in the reactions of the victims. In addition, other individual factors such as economic status, personality, and life experience were not explored as potential factors affecting the likelihood of experiencing sexual harassment, response style, or impact on factors such as job satisfaction or mental health.

We focused on nurses’ perceptions of the general phenomenon of sexual harassment in nursing. Speaking with nurses who have first hand experiences could add richness of detail to the contextual and circumstantial factors surrounding the incidents. A broader community-based study on societal attitudes about the reputation of the nursing profession could provide additional depth and detail on the enabling factors. Further research with Sri Lanka’s small population of male nurses could also provide insight into risk and protective factors related to gender and power relations in society and workplaces. There is significant scope for future research.

## Conclusion

By exploring nurses’ perceptions of sexual harassment in a large hospital in Sri Lanka, this study draws attention to a rarely acknowledged issue of concern for the health workforce in the country. Continued research into policies and strategies for preventing and addressing sexual harassment in healthcare settings is required. The nurses we consulted feel that there is not yet a transparent system for reporting and addressing incidents of sexual harassment, and this needs to change.

Increasing awareness on sexual harassment in the Sri Lankan healthcare setting puts this issue on the health policy agenda. Many of the negative health and social consequences associated with sexual harassment can be addressed by improved education, accountability, policy, prevention, and intervention strategies. Addressing incidents of sexual harassment in hospital settings is essential for ensuring an effective workforce and high-quality health services.

## Paper context

This is the first paper to explore sexual harassment in Sri Lanka’s health sector. Female nurses perceived a variety of forms of sexual harassment in a hospital setting, perpetrated by male patients and co-workers. They highlighted inhibitors to reporting the incidents and the negative impact on societal perceptions of the nursing profession. Improved education, accountability, prevention, and intervention strategies are required to ensure the health of nurses and quality and efficiency of the health system.
